# R/Aging Parents: Seeking Advice on Fall Prevention Technologies on Reddit

**DOI:** 10.1093/geroni/igaf122.2639

**Published:** 2025-12-31

**Authors:** Jaahnvi Patel, Shannon Mejia

**Affiliations:** University of Illinois Urbana-Champaign, Champaign, Illinois, United States; University of Illinois Urbana-Champaign, Champaign, Illinois, United States

## Abstract

The first generation of Reddit users are now middle-aged and their parents are aging. Accidental falls are a common and potentially life-changing event that can signal to children that their parents are aging. An open question is the role that the Reddit community plays in supporting adult children as they navigate fall prevention for their aging parents. This research conducts content analysis of the AgingParents subreddit to examine how caregivers seek and receive advice on fall prevention technology. The sample included 113 threads within the AgingParents subreddit that were posted since February 2023. A codebook was developed to categorize user posts based on the relationship of the original poster (OP) to the older adult (OA), their living situation, level of care needs, and use of fall prevention technology. We coded for key concerns, technology adoption, and types of support sought and received. Responses were analyzed to determine the types of support given, including emotional, informational, tangible, belonging, and technology-related support, as well as misinformation or conflicting advice. The findings suggest that adult children frequently seek advice on fall prevention technologies, discussing their usability and their parents’ resistance. Caregivers also expressed emotional burden and family decision-making challenges, which could contribute to motivations for seeking support on Reddit. The study highlights Reddit’s role as a support network and provides key insights into how Reddit functions as a support network for caregivers and highlights areas for improving fall prevention strategy discussion.

